# The Use of Oritavancin as Consolidation Therapy for Infective Endocarditis Caused by *Enterococcus* spp.

**DOI:** 10.1093/ofid/ofaf416

**Published:** 2025-07-30

**Authors:** Federico Frondizi, Gabriele Maria Leanza, Marta Chiuchiarelli, Francesca Catania, Flavio Sangiorgi, Francesca Giovannenze, Eleonora Taddei, Enrica Tamburrini, Carlo Torti, Giancarlo Scoppettuolo

**Affiliations:** Sezione Malattie Infettive, Dipartimento di Sicurezza e Bioetica, Università Cattolica S. Cuore, Roma, Italia; Sezione Malattie Infettive, Dipartimento di Sicurezza e Bioetica, Università Cattolica S. Cuore, Roma, Italia; Fondazione Policlinico Universitario A. Gemelli IRCCS, Roma Italia- Dipartimento di Scienze Mediche e Chirurgiche- UOC Malattie infettive, Rome, Italy; Sezione Malattie Infettive, Dipartimento di Sicurezza e Bioetica, Università Cattolica S. Cuore, Roma, Italia; Sezione Malattie Infettive, Dipartimento di Sicurezza e Bioetica, Università Cattolica S. Cuore, Roma, Italia; UOC Malattie Infettive ad Interesse Chirurgico, Clinical Infectious Diseases Department, National Institute for Infectious Diseases Lazzaro Spallanzani IRCCS, Rome, Italy; Fondazione Policlinico Universitario A. Gemelli IRCCS, Roma Italia- Dipartimento di Scienze Mediche e Chirurgiche- UOC Malattie infettive, Rome, Italy; Fondazione Policlinico Universitario A. Gemelli IRCCS, Roma Italia- Dipartimento di Scienze Mediche e Chirurgiche- UOC Malattie infettive, Rome, Italy; Sezione Malattie Infettive, Dipartimento di Sicurezza e Bioetica, Università Cattolica S. Cuore, Roma, Italia; Fondazione Policlinico Universitario A. Gemelli IRCCS, Roma Italia- Dipartimento di Scienze Mediche e Chirurgiche- UOC Malattie infettive, Rome, Italy; Sezione Malattie Infettive, Dipartimento di Sicurezza e Bioetica, Università Cattolica S. Cuore, Roma, Italia; Fondazione Policlinico Universitario A. Gemelli IRCCS, Roma Italia- Dipartimento di Scienze Mediche e Chirurgiche- UOC Malattie infettive, Rome, Italy; Fondazione Policlinico Universitario A. Gemelli IRCCS, Roma Italia- Dipartimento di Scienze Mediche e Chirurgiche- UOC Malattie infettive, Rome, Italy

**Keywords:** endocarditis, *Enterococcus*, oritavancin

## Abstract

Oritavancin is emerging as a potential alternative to standard antibiotic regimens in the treatment of infective endocarditis caused by gram-positive bacteria, though evidence remains limited. We hereby report 7 cases of enterococcal endocarditis treated with oritavancin as consolidation therapy, resulting in 6 cures and 1 relapse.

Oritavancin is a long-acting lipoglycopeptide approved for treating skin and soft tissue infections caused by gram-positive bacteria, including drug-sensitive and drug-resistant species such as *Staphylococcus aureus* (methicillin-susceptible *S. aureus* [MSSA], methicillin-resistant *S. aureus* [MRSA], vancomycin-intermediate *S. aureus* [VISA], heterogeneous VISA [hVISA], vancomycin-resistant *S. aureus* [VRSA]), streptococci, and enterococci—both vancomycin-variable and -resistant strains [1, 2].

Although it has been suggested as a promising consolidation strategy for conditions requiring long-course antibiotic therapy, such as infective endocarditis [3, 4], current evidence is limited to isolated case reports [5, 6] and a single case series [7]. Additionally, while recent studies have already claimed the potential role of dalbavancin in these therapeutic contexts [8, 9], several concerns have been raised regarding its use for *Enterococcus* spp. infections: In vitro studies [10] pointed out suboptimal efficacy against VRE, and real-world clinical experiences [11, 12] have shown challenges in treating *Enterococcus faecalis*.

Here we present a case series examining the role of oritavancin as consolidation therapy for infective endocarditis.

## CASE DESCRIPTION

As noted in Tables 1 and 2, we retrieved 7 cases of infective endocarditis treated with oritavancin at Policlinico Universitario “A. Gemelli” in Rome: 6 men and 1 woman, with a median age (range) of 75 (61–88) years.

**Table 1.** *Overview of E. faecium* Cases

	#1	#2	#3
Date of IE diagnosis, month and year	May 2024	May 2024	November 2023
Age, y	66	61	88
Sex	M	M	F
Comorbidities	Hypertension, colonic diverticulosis, and previous endocarditis due to *Streptococcus gordonii*, treated with antibiotic therapy and followed by aortic valve replacement	Hypertension and atrial fibrillation	Severe aortic stenosis, hypertrophic-infiltrative cardiopathy, hypertension, colonic diverticulosis, monoclonal gammopathy
BMI, kg/m^2^	21.97	25.10	25.39
Vancomycin resistance	Yes	No	Yes
IE type	PVE	NVE	NVE
Valve affected	Aortic	Mitral–aortic	Mitral–aortic
Imaging	Diffuse aortic prosthesis thickening	Diffuse remodeling of the aortic valve associated with a mitro–aortic pseudoaneurysm	Multiple subcentimetric vegetations
Embolizations	Spondylodiscitis C6–C7	Splenic and cerebral embolizations	No
Surgery	No	Yes; positive valve culture	No
Colonoscopy	Yes; negative for neoplasms	Not performed	Not performed
Initial antibiotic therapy	Linezolid + daptomycin	Daptomycin + ceftaroline Fosfomycin + teicoplanin	Linezolid + daptomycin
Time to oritavancin, d since IE diagnosis	30	27	35
Oritavancin dosing regimen	1200 mg × 2 (1 wk apart)	1200 mg × 2 (1 wk apart)	1200 mg (single dose)
Concomitant oral therapy	No	No	No
Outcome	Alive at 12 mo; IE relapse at 8 mo treated with valve replacement	Alive at 12 mo with no relapses	Death at 3 mo due to non-IE-related causes
Adverse events	No	No	No

Abbreviations: BMI, body mass index; CIED, cardiac implantable electronic device; CRT-D, cardiac resynchronization therapy with defibrillator; NVE, native valve endocarditis; PVE, prosthetic valve endocarditis.

**Table 2.** Overview of *E. faecalis* Cases

	#4	#5	#6	#7
Date of IE diagnosis, month and year	November 2023	May 2024	April 2024	June 2024
Age, y	75	77	79	63
Sex	M	M	M	M
Comorbidities	Ischemic heart disease, hypertension	Ischemic heart disease, atrial fibrillation, CRT-D wearer	Ischemic heart disease, hypertension, mild cognitive impairment	Chronic obstructive bronchitis, atrial fibrillation, monoclonal gammopathy
BMI, kg/m^2^	24.03	20.42	22.49	20.83
Vancomycin resistance	No	No	No	No
IE type	PVE	NVE with CIED involvement	PVE	NVE
Valve affected	Aortic	Pulmonary	Mitral	Mitral
Imaging	Diffuse aortic prosthesis thickening with FDG uptake on PET-CT, without local complications	Vegetation of 20 × 10 mm, without local complications	Diffuse mitral prosthesis thickening with FDG hyperuptake on PET-CT, without local complications	Presence of a 22-mm vegetation and perforation of the mitral valve
Embolizations	No	Lung embolization	Splenic embolization	Lung embolizations
Surgery	No	No	No	Yes; negative valve culture
Colonoscopy	Yes; negative for neoplasms	Yes; tubulovillous adenoma removed endoscopically	Not performed	Yes; negative for neoplasms
Initial antibiotic therapy	Ampicillin + ceftriaxone	Ampicillin + ceftriaxone	Ampicillin + ceftriaxone	Ampicillin + ceftriaxone
Time to oritavancin	23	27	23	82
Oritavancin dosing regimen	1200 mg (single dose)	1200 mg × 2 (1 wk apart)	1200 mg (single dose)	1200 mg (single dose)
Concomitant oral therapy	Yes (amoxicillin 1 g every 8 h; therapy is ongoing)	Yes (amoxicillin 1 g every 8 h; therapy is ongoing)	Yes (amoxicillin 1 g every 8 h; therapy is ongoing)	No
Outcome	Alive at 12 mo with no relapses	Alive at 12 mo with no relapses	Alive at 12 mo with no relapses	Alive at 12 mo with no relapses
Adverse events	No	No	No	No

Abbreviations: BMI, body mass index; CIED, cardiac implantable electronic device; CRT-D, cardiac resynchronization therapy with defibrillator; NVE, native valve endocarditis; PVE, prosthetic valve endocarditis.

The cases included prosthetic valve endocarditis (PVE; n = 3), native valve endocarditis (NVE; n = 3), and cardiac implantable electronic device–related endocarditis (CIED-IE; n = 1), with involvement of the aortic (n = 2), mitral (n = 2), mitral–aortic (n = 2), and pulmonary (n = 1) valves. The index pathogens were *Enterococcus faecalis* (n = 4) and *Enterococcus faecium*, including 2 vancomycin-resistant (VRE) and 1 vancomycin-susceptible (VSE) strain.

Five patients were deemed ineligible for surgery, while only 2 underwent valve replacement.

Regarding the 3 cases of PVE, none underwent cardiac surgery; 2 transitioned to oral long-term amoxicillin therapy, while 1 discontinued antibiotic treatment.

Concerning the case of native pulmonary valve endocarditis associated with CIED infection, chronic suppressive therapy with amoxicillin was continued due to the patient's poor performance status, which contraindicated device extraction.

In those cases where long-term antibiotic therapy was adopted, oritavancin was used as the tail end of the initial intravenous regimen to complete a total of at least 4–6 weeks of effective parenteral therapy [13]. The median time from infective endocarditis diagnosis to oritavancin administration (range) was 27 (23–82) days, with initial therapy tailored to the pathogen. The *E. faecalis* cases were treated with ampicillin plus ceftriaxone. The VRE cases received initial therapy with daptomycin plus linezolid, while the VSE case was initially managed with daptomycin and ceftaroline. However, due to the development of daptomycin-related muscle toxicity, therapy was subsequently switched to fosfomycin and teicoplanin.

Microbiological clearance was achieved before transitioning to long-acting therapy, and informed consent was obtained from all patients before oritavancin administration.

Three patients received 2 doses of 1200 mg of oritavancin administered 1 week apart, either before discharge or in an outpatient setting, while 4 received a single 1200-mg dose before discharge.

Overall, 5 patients remained alive and relapse-free at 12 months following diagnosis of endocarditis.

As detailed in Table 1, 1 patient with a diagnosis of NVE caused by *vancomycin-resistant E. faecium*, who also suffered from chronic severe congestive heart failure, was readmitted 2 months after discharge and died from pulmonary edema unrelated to IE.Additionally, 1 patient with VRE prosthetic valve endocarditis, who neither underwent cardiac surgery nor received long-term oral therapy, experienced a relapse 8 months after the initial diagnosis and required a second valve replacement.

Notably, no infusion-related reactions or adverse events were reported. Furthermore, no alterations in prothrombin time or activated partial thromboplastin time were observed.

## DISCUSSION

Given its spectrum of activity and pharmacokinetic properties, oritavancin represents an interesting alternative for consolidation therapy in infective endocarditis. However, clinical trials and large observational studies on its use in this setting are lacking.

In this study, oritavancin was introduced within 3–5 weeks of diagnosis in most patients (6 out of 7) as a continuation therapy after initial antibiotic induction. Notably, in the 3 cases requiring chronic suppressive therapy, early administration of oritavancin (within 3–4 weeks) enabled earlier hospital discharge. These findings support the potential of oritavancin to reduce hospital length of stay, particularly in patients requiring prolonged antibiotic regimens, in line with evidence from studies of other long-acting lipoglycopeptides such as dalbavancin, which has proven successful in facilitating earlier discharge [14].

As previously mentioned, among the 7 cases reported, only 1 patient experienced a relapse of endocarditis. This case involved a 66-year-old patient with a diagnosis of possible PVE caused by VRE who was initially not considered a candidate for valve replacement surgery. Due to the limited susceptibility of the microorganism to oral antibiotic agents and the inability to administer multiple doses of oritavancin guided by therapeutic drug monitoring (TDM), a single dose of oritavancin was administered following 4 weeks of intravenous antibiotic therapy to consolidate treatment of infective endocarditis. The patient was then discharged with close clinical and radiological follow-up, without subsequent antibiotic therapy as a suppressive strategy. Eight months after the initial diagnosis, the patient was readmitted due to a recurrence of VRE bacteremia, with identification of a new endocarditic vegetation on the prosthetic valve that was previously affected. As a result, the patient underwent valve replacement surgery.

While dalbavancin has been more extensively evaluated and proposed as sequential therapy for infective endocarditis, particularly in staphylococcal infections [8, 9, 15, 16], its use in enterococcal endocarditis remains controversial. Suárez et al. [12] reported 5 treatment failures after dalbavancin, 4 of which involved vancomycin-susceptible *Enterococcus* spp., raising concerns about dalbavancin’s reliability in this context. More recently, a multicenter study involving 98 cases of endocarditis treated with dalbavancin as consolidation therapy reported a recurrence rate of 8%, with 7 cases caused by *E. faecalis* and 1 by *E. faecium* [11].

In apparent contrast to what has been reported with dalbavancin, our data further support the growing interest in oritavancin as an alternative long-acting glycopeptide, particularly due to its activity against both vancomycin-susceptible and -resistant enterococci.

In 2024, Giuliano et al. [6] described a case of native tricuspid valve endocarditis caused by *E. faecium* in a 35-year-old intravenous drug user successfully treated with oritavancin after 4 weeks of intravenous vancomycin. In contrast, our case series expands the application of oritavancin to an older population (all patients were over 60 years of age), reflecting the shifting epidemiology of enterococcal endocarditis [17, 18]. In these elderly patients, often deemed unfit for surgery due to reduced physiological reserve and higher comorbidity burden, oritavancin could offer a valuable treatment strategy. Its potential to allow early hospital discharge may reduce both infective and noninfective complications, offering a lower pharmacological toxicity profile compared with other oral antibiotic treatment options (eg, linezolid for VRE endocarditis). Although promising in these contexts, the role of oritavancin in chronic suppressive therapy for VRE infections remains unexplored and would require TDM to ensure safety and efficacy [19].

This study has several limitations. The first is the lack of in vitro susceptibility data for microbiological isolates to oritavancin, along with the absence of TDM to guide administration, although no validated pharmacokinetic/pharmacodynamic targets currently exist to support oritavancin’s clinical use. Furthermore, in 1 patient, oritavancin was initiated with a considerable delay (82 days since diagnosis), limiting the interpretability of its therapeutic impact in this case of endocarditis. Notably, in 3 cases, oritavancin was also prescribed in combination with oral antibiotic therapy for a chronic suppressive treatment purpose, potentially confounding the assessment of oritavancin's impact on clinical outcomes.

## CONCLUSIONS

To our knowledge, this is the largest case series focusing on oritavancin as sequential therapy for infective endocarditis. Our findings suggest that oritavancin may offer a safe and effective treatment consolidation strategy, one that is particularly valuable in elderly patients with comorbidities and polypharmacy, in whom prolonged hospitalization and complex antibiotic regimens are often unfeasible. Moreover, these data may help guide future studies to address the current evidence gap on the clinical efficacy of oritavancin in this setting. Robust clinical trials and larger observational studies incorporating susceptibility testing are warranted to better define the role of oritavancin in the management of enterococcal endocarditis.
